# Local Administration of Soluble CD40:Fc to the Salivary Glands of Non-Obese Diabetic Mice Does Not Ameliorate Autoimmune Inflammation

**DOI:** 10.1371/journal.pone.0051375

**Published:** 2012-12-26

**Authors:** Nienke Roescher, Jelle L. Vosters, Zhenan Lai, Toshimitsu Uede, Paul P. Tak, John A. Chiorini

**Affiliations:** 1 Molecular Physiology and Therapeutics Branch, National Institute of Dental and Craniofacial Research, National Institutes of Health, Bethesda, Maryland, United States of America; 2 Division of Clinical Immunology & Rheumatology, Academic Medical Center/University of Amsterdam, Amsterdam, The Netherlands; 3 GlaxoSmithKline, London, United Kingdom; 4 Division of Molecular Immunology, Institute for Genetic Medicine, Hokkaido University, Sapporo, Japan; University of Amsterdam Academic Medical Center, The Netherlands

## Abstract

**Objective:**

CD40–CD154 (CD40 ligand) interaction in the co-stimulatory pathway is involved in many (auto)immune processes and both molecules are upregulated in salivary glands of Sjögren’s syndrome (SS) patients. Interference within the CD40 pathway has ameliorated (auto)inflammation in a number of disease models. To test the potential role of the CD40 pathway in loss of gland function and inflammation in SS, an inhibitor of CD40-CD154 interaction was overexpressed in the salivary glands (SGs) of a spontaneous murine model of SS; the Non-Obese Diabetic (NOD) mouse.

**Materials and Methods:**

At different disease stages an adeno associated viral vector encoding CD40 coupled to a human Fc domain (CD40:Fc) was injected locally into the SGs of NOD mice. Delivery was confirmed by PCR. The overall effect on local inflammation was determined by assessment of the focus score (FS), quantification of infiltrating cell types, immunoglobulin levels, and microarray analysis. The effect on SG function was determined by measuring stimulated salivary flow.

**Results:**

CD40:Fc was stably expressed in the SG of NOD mice, and the protein was secreted into the blood stream. Microarray analysis revealed that expression of CD40:Fc affected the expression of many genes involved in regulation of the immune response. However, FS, infiltrating cell types, immunoglobulin levels, and salivary gland output were similar for treated and control mice.

**Discussion:**

Although endogenous CD40 is expressed in SG inflammatory foci in the SG of NOD mice, the expression of soluble CD40:Fc did not lead to reduced overall inflammation and/or improved salivary gland function. These data indicate possible redundancy of the CD40 pathway in the SG and suggests that targeting CD40 alone may not be sufficient to alter the disease phenotype.

## Introduction

The inflammatory foci observed in the salivary gland (SG) of non-obese diabetic (NOD) mice resemble the foci comprised of mononuclear cells seen in SGs of patients with Sjögren’s syndrome (SS) [1]. SS is a chronic inflammatory autoimmune disease mainly affecting the lachrymal and salivary glands (LG and SG respectively). It is very common with an estimated prevalence of 0.5%–2% in the general population (of which 9 out of 10 is female). The disease is currently incurable and the symptoms are challenging to manage. The local autoimmune process is characterized by the influx of T cells and to a lesser degree B cells, and a variety of other immune cells that accumulate in the secretory glands and reorganize over time [2]. It is unclear what initiates the inflammatory process, but the upregulation of co-stimulatory-, adhesion- and antigen-presenting molecules is thought to play a role in the recruitment and the organization of inflammatory cells in the SG of both patients and mice. The engagement of the co-stimulatory molecules CD40, belonging to the tumor necrosis factor (TNF) receptor superfamily, and its ligand, CD154 is known to induce B cell activation and maturation, immunoglobulin isotype switching and the secretion of pro-inflammatory cytokines such as interleukin (IL)-1, IL-6, IL-12 and interferon (IFN)-γ [3]. CD154 is expressed on CD4^+^ T cells, but can also be found on a variety of non-lymphoid cells. CD40 can also be found on many cell types such as B cells, endothelial cells, dendritic cells and monocytes [4]. In the SG of SS patients, CD40 is detected on epithelial cells, lymphocytes and endothelial cells [5], [6]. CD40 is upregulated on epithelial cells *in vitro* when stimulated with cytokines such as IFN-γ and IL-1β [7]. In addition, stimulation through CD40 leads to activation of SG epithelial cells as indicated by upregulation of intercellular adhesion molecule type 1 (ICAM-1) [8]. CD154 can be found in the clustered infiltrating cells [5], [6].

The interaction of CD40 and CD154 has been implicated in a number of diseases such as arthritis, cancer, atherosclerosis, lupus nephritis, and acute or chronic graft-versus-host disease [4]. Blocking and/or interfering with this specific co-stimulatory pathway has been studied previously in animal models of transplant rejection [9], [10], [11], diabetes [12] and experimental autoimmune encephalomyelitis (EAE) [13] with improved clinical outcome. The effect of altered CD40-CD154 interaction has not been studied in animal models of SS. Therefore, we tested the effect of overexpression of soluble CD40 on the SG inflammation of NOD mice at 3 different stages of the disease: at 8 weeks of age when the majority of NOD mice have not yet developed focal inflammation, at this age endogenous CD40 is not detected in SG of the NOD mice who do not have infiltrates; At 10 weeks when focal inflammation is clearly present and CD40 can be found in the early SG foci; and at 16 weeks in a more advanced disease stage when CD40 is strongly upregulated within infiltrates [2]. Although expression of CD40:Fc lead to changes in the SG transcriptome it did not result in a reduction in inflammation nor in improved salivary gland function.

## Results

### *In vitro* and *in vivo* Expression of CD40:Fc

**Figure 1:**
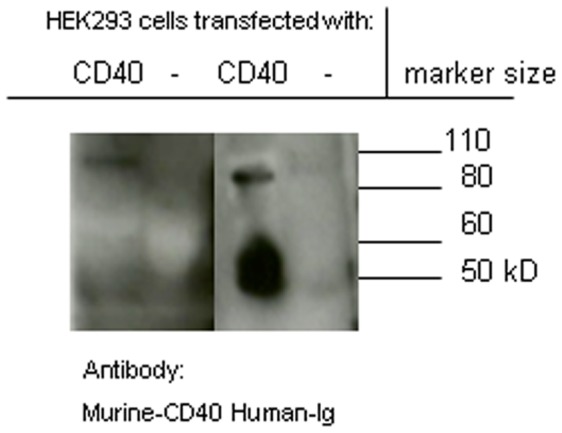
Western blot of supernatant of HEK293 transfected cells showing CD40:Fc expression. HEK293 cells were transduced with AAV2-CD40:Fc or were mock transduced. The fusion protein was detected by anti murine-CD40 (left panel) and anti-human-Fc (right) and showed a band ∼90 kD (size of non-reduced dimer) and a ∼45 kD (reduced monomer) band.

Murine CD40 coupled to the constant region of human immunoglobulin (Fc) under the regulation of CMV promoter was cloned into a recombinant AAV vector plasmid and was transduced into HEK293 cells. After 48 hours (hrs), supernatant was collected and tested for expression by western blot and ELISA. The secreted CD40:Fc protein migrated as expected as a dimer under non-reduced conditions (90 kD) and as a monomer of approximately 45 kD under reduced conditions. Furthermore, the product could be detected by an anti-human-Ig and an anti-murine CD40 antibody (Figure 1). In an ELISA, 2.86 µg/ml protein was detected by anti-murine CD40 in the supernatant of transduced cells. The fusion protein could also be detected by anti-human Ig, suggesting full protein expression (data not shown). This data is in agreement with previous studies that have shown expression as well as biologic activity of this fusion protein *in vivo* [9], [11].

NOD mice express CD40 positive focal infiltrates in the SG that start to appear between 8–10 weeks of age and become prominent at 12 weeks of age [2]. To study the effect of soluble CD40 on different stages of autoimmune sialadenitis, the CD40:Fc vector was infused into the SG of NOD mice at the age of 8, 10 and 16 weeks (at the initiation of immune cell influx, during the accelerated phase of immune cell influx and at a more established disease phase respectively). AAV2 is known to stably transduce epithelial cells in the salivary gland and provokes by itself only a minimal immune responses [14]. Furthermore, mice treated with control AAV-constructs encoding for LacZ show similar degrees of inflammation in the SG when compared to untreated mice [2]. At the age of 16 or 20 weeks, mice were euthanized and Q-PCR was performed to confirm local delivery of the vector to the SG and vector was detected in SGs of the treated mice but not in the liver (data not shown). Saliva, serum and protein homogenates from CD40:Fc treated and control mice was analyzed for CD40 expression and showed expression in serum (∼10 ng/ml) of treated mice while it was not detected in control mice (<3.5 pg/ml). The fusion protein was not detected in saliva or protein homogenates. In the i.m. treated mice CD40:Fc was not detectable (data not shown).

### Changes in the Transcriptome of CD40:Fc Treated Mice

**Figure 2:**
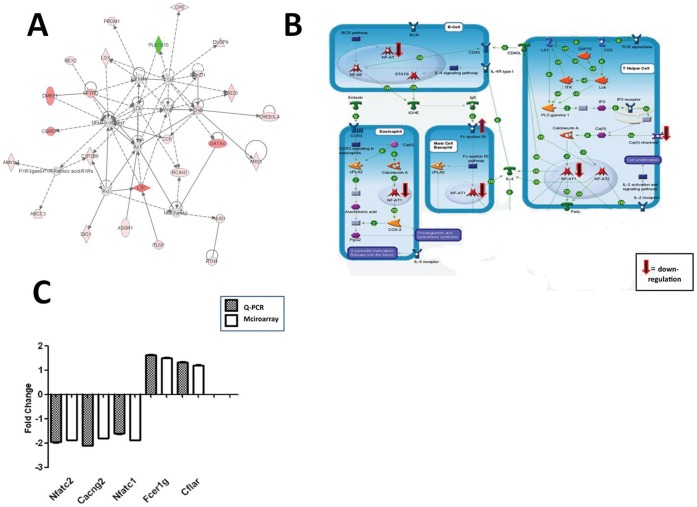
Network based analysis of genes affected by CD40:Fc treatment. Microarray analysis was performed on SG of CD40:Fc treated and control mice (n = 4 each, treatment at 10 weeks, end of study at 20 weeks) and gene changes greater than 2 fold were used in the analysis and compared for biological functional pathways or biomarkers by an Ingenuity Pathway analysis (IPA). (**A**) The pathway of NF-κB and TGF-β was affected in CD40:Fc treated mice. Green colored shapes indicates upregulated genes, red colored shapes downregulated; gray colored shapes genes in the pathway that were not differentially expressed. Color intensity reflects the degree of differential expression. Triangles represent phosphatases; horizontal diamond, peptidases; vertical diamond, enzymes; horizontal oval, transcription factors; vertical oval, transmembrane receptors; trapezoid, transporters; circle, other type of protein. Dotted lines indicate an indirect interaction; solid line, direct interaction, solid arrow head indicates “acts on”. (**B**) Metacore analysis of pathways linking differentially expressed genes to immune pathways. The differentially expressed genes are indicated along with the direction of change in expression. (**C**) Quantitative-PCR of selected genes shows agreement with microarray results. The results obtained using the microarray platform were validated by examining the correlation between the expression levels in the microarray and qPCR results obtained for a subset of genes.

Microarray analysis was performed on the mRNA derived from the SGs of mice treated at 10 weeks and euthanized at 20 weeks and this revealed a number of changes in the transcriptome of the CD40 treated mice compared with the LacZ treated mice. Ingenuity pathway analysis of either all statistically significant differentially expressed genes or just those over 2 fold, identified the gene expression/immune response network as a key network for the differentially expressed genes. Central nodes in this network include the transcription factor nuclear factor (NF)-κB complex and transforming growth factor (TGF)-β. Many of the genes surrounding these nodes were downregulated in the CD40:Fc treated mice compared with the LacZ group suggesting decreased activation in the CD40:Fc group (Figure 2A). Complementary analysis using genego (metacore), which uses an independent pathway analysis database identified additional changes linked to lymphocyte function including NFAT, voltage dependent calcium channel, high affinity IgE receptor, and the apoptosis regulator CFLAR (Fig. 2B). Quantitative PCR analysis showed results in agreement with the microarray gene expression in both genes that were up regulated as well as down regulated in the CD40:FC treated mice compared with control mice (Fig. 2C).

In total, 558 Genes were identified as statistically significantly differentially expressed in CD40 mice compared with controls with 137 genes that were up- and 421 genes that were down-regulated. Table 1 shows genes that were found to be more than 10 fold down or more than 5 fold up regulated.

**Table 1:** Up or down regulated genes in salivary glands of CD40:Fc treated mice versus control mice.

DOWN		UP	
GENE	FOLD CHANGE	GENE	FOLD CHANGE
AK086138	24,9	A430089I19Rik	8,2
AK052096	22,3	0610040A22Rik	5,5
ENSMUST00000103537	20,1		
Zfp59	20,0		
Dio1	18,2		
2310035C23Rik	17,8		
BB473918	16,4		
AB070552	16,0		
Ltf	15,7		
Slc38a5	13,9		
Dmbt1	12,9		
Gata4	12,4		
Tmc5	12,2		
Xkr8	12,1		
Gsta3	12,0		
Elavl4	11,9		
Nkx2-3	11,4		
Klra5	10,9		

Top gene expression changes following, t-test plus Benjamin correction for false discovery. Expression changes greater than 10 fold down or 5 fold up are shown.

The function of many of the most significantly differentially expressed genes are unknown. However, amongst the list of downregulated genes in the CD40:Fc treated group compared with control mice were a few genes of particular interest. Dio1 (downregulated 18,2 times) is the gene for type 1 deiodinase (D1), which provides the majority of the circulating thyroid hormone T3 in vertebrates [15]. T3 is known to be downregulated during (chronic) inflammation, possibly related to the expression of pro-inflammatory cytokines such as IL-6 and IL-1 [16]. Another gene found to be downregulated in the CD40:Fc treated mice was Ltf, encoding for lactoferrin (Ltf), a iron-binding glycoprotein which has anti-microbial properties and is involved in inflammation, mainly in innate immunity [17]. It can activate macrophages and induce IL-6 [18]. Dmbt1 (deleted in malignant brain tumors 1) is downregulated 12.9 fold and is involved in the mucosal immune defense [19]. The transcription factor GATA4, which is involved in TGF-β signaling and is upregulated in patients with inflammatory bowel disease, was also downregulated. Together these results suggest that overexpression of CD40:Fc is able to decrease the pro-inflammatory/autoimmune environment associated with SG pathology in the NOD mouse.

### Focus Scores and Composition of Leukocyte Infiltrates are not Affected after CD40:Fc Treatment

**Figure 3:**
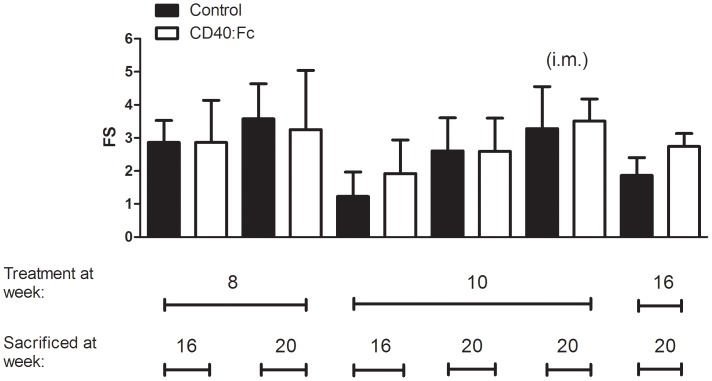
Focus score (FS) of salivary glands (SG) of non-obese diabetic (NOD) mice treated with CD40:Fc at different ages. FS was determined for each group of CD40:Fc treated mice and compared with control mice (n = 8–10 mice per group). Shown is the average FS per group. Error bar = standard deviation (SD), i.m. = intramuscular.

**Figure 4:**
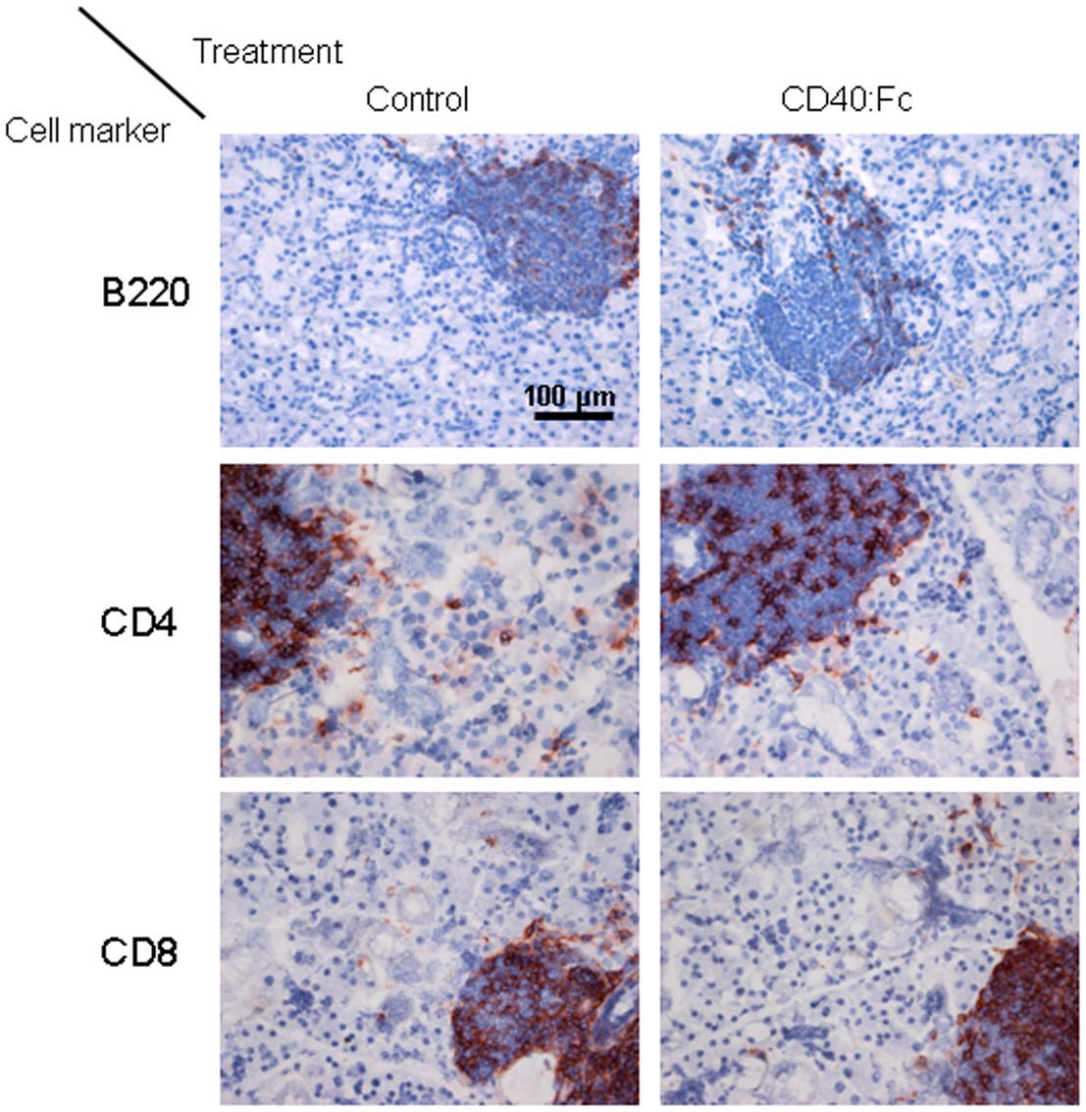
T and B cell presence in SG of 20 week old CD40:Fc or control treated NOD. Salivary gland (SG) tissue was cut in sections and stained with anti-CD4, anti-CD8 and anti-B220 antibodies. Representative photos (group n = 8 mice each) of mice treated at 10 weeks are shown at the end of the study at 20 weeks. B220^+^ = B cells, CD4^+^ = Th cells and CD8^+^ = cytotoxic T cells.

In order to test if the changes in the gene profile in CD40:Fc treated mice had an effect on the SS-like phenotype in the NOD mice, the SG associated FS was quantified in the CD40:Fc treated and control mice. Infiltrates were detected in both the CD40:Fc and LacZ treated groups, however no differences in scores were measured between the two groups (Figure 3). In addition, we quantified a number of different immune cells that normally form the focal infiltrates [2], CD4^+^ and CD8^+^ T cells, CD19^+^ and B220^+^ B cells, CD11c^+^ dendritic cells and CD49b^+^ natural killer (NK) cells or diffusely infiltrate the SG (CD68^+^ macrophages) and found no statistically significant differences in the presence of these subtypes of cells (shown are B (B220^+^) and T (CD4^+^ and CD8^+^) cells, Figure 4).

### CD40:Fc Treatment does not Change Salivary Flow

**Figure 5:**
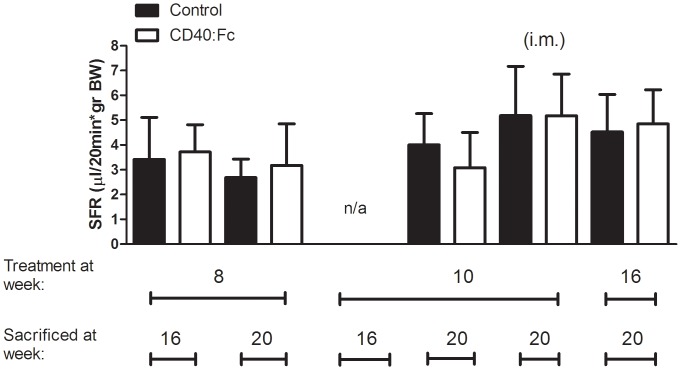
Stimulated salivary flow of CD40:Fc treated and control non-obese diabetic mice. At the indicated age, mice were anesthetized and given pilocarpine to stimulate salivary flow and saliva was collected. Salivary flows corrected for body weight (SFR) of the different treatment groups are shown as a mean+standard deviation (SD; n = 8–10 per group, i.m. = intramuscular, n/a = not available).

One of the clinical hallmarks of SS is the loss of salivary flow. NOD mice show reduced salivary flow rates (SFR) with increasing age; in our facility the SFR starts to decline after the age of 16 weeks. This reduction in SFR often coincides with focal inflammation, but focal inflammation and SFR are not directly correlated [2]. To study the effect of CD40:Fc on SFR, we measured the pilocarpine stimulated salivary flow 5 days prior to the end of the study for all the mice treated at the different time intervals. As shown in figure 5, stimulated SFR was unaffected by CD40:Fc treatment when compared with controls.

### Immunoglobulin Levels were not Affected by CD40:Fc Treatment

CD40 stimulation leads to isotype switching and antibody production of B and plasma cells [20]. Despite the changes in the expression of genes related to the immune response and the known role of CD40 in B cell function, the number of B and plasma cells in the SG was unaffected after treatment. Immunoglobulin (Ig) A, G and M levels were measured in salivary gland homogenates and serum and no change was observed for the different Ig-types in either serum or SG homogenates for CD40:Fc compared to LacZ treated mice (data not shown).

## Discussion

CD40 and CD154 are upregulated in SG of SS patients [5], [6] and in NOD mice developing a SS-like phenotype [2]. Interfering with the CD40-CD154 interaction by monoclonal antibodies, soluble ligands or antisense oligonucleotides has been successfully applied to mouse models of autoimmune disease, such as diabetes, transplant rejection, and EAE, but has not been studied before in animal models of SS. Therefore we tested whether the expression of soluble murine CD40 coupled to an Fc domain of human IgG locally in the SG of NOD mice could affect the SS like phenotype that develops in these mice. We show that local overexpression of CD40:Fc in the SG of NOD mice led to changes in the expression of genes involved in inflammation and immunity, but this did not result in reduced inflammation within the SG or improved salivary gland function.

We administered CD40:Fc to the SG of NOD mice of different ages/disease stages with the aid of an AAV2 vector. Local delivery of the vector was confirmed by PCR of the SG tissue. We detected the CD40:Fc fusion protein in serum, but not in saliva. This indicates that the CD40:Fc protein was properly processed for secretion via the constitutive secretory pathway of the SG. CD40:Fc was not detected in the SG protein homogenates from the treated mice. This was possibly due to high background and cross-reactivity with endogenous (murine)CD40 and immunoglobulins which may have masked proper detection in our tests. After i.m. administration, the fusion protein could not be detected in serum. This is in agreement with data showing that AAV2 transduced muscle cells are poor producers of protein [21] and supports the use of the SG as a bio-producer of immunomodulatory proteins.

After CD40:Fc treatment, we found a number of differentially expressed SG mRNAs in the CD40:Fc treated mice compared with control (LacZ) treated mice. Analysis identified the gene expression/immune response network as a key network for the differentially expressed genes with NF-κB, NFAT, and TGF-β at the center. NF-κB is a major transcription factor regulating genes responsible for both the innate and adaptive immune response. Upon activation of either the T- or B-cell receptor in combination with CD40, NF-κB becomes activated through specific signaling pathways [22], [23], [24], [25]. TGF-β is involved in this pathway and can regulate NF-κB and CD40 under different circumstances [26], [27], [28], [29]. In addition, microarray analysis revealed that several down-regulated genes in the CD40:Fc treated mice were involved in inflammation. Finding alterations in the NF-κB network and these gene changes supported the notion that CD40:Fc is functional and able to affect the local immunological environment.

In NOD mice, endogenous CD40 expression can be found in SG in and around aggregates which start to form between 8–10 weeks, but are most prominent from 12 weeks of age onwards. Unlike ICAM-1, which is also involved in the co-stimulatory pathway, CD40 is not expressed prior to the influx of immune cells [2]. NOD mice are reported to have changes in gland activity as early as 6 weeks of age [1]; however cannulation at this age is technically not possible due to the anatomically immature state of the salivary ducts. Previously we studied the effect of soluble (s)ICAM-1:Fc on inflammation of the SG in NOD. ICAM-1 was found to be expressed in epithelial and endothelial structures before infiltrates formed. We found that treatment with sICAM-1:Fc at 8 weeks, but not at 10 weeks, reduced overall inflammation based on FS. In contrast, late treatment led to a higher number of infiltrated CD4^+^ and CD8^+^ T cells. sICAM-1:Fc expression did not prevent the loss of salivary function in either treatment group [30].

There are a number of possible reasons for the lack of therapeutic effect. First CD40, although present in SG of patients and mice with autoimmune sialadenitis, may not be crucial for immune cell infiltration. ICAM-1 is a molecule involved in co-stimulation but also in cell-adherence and this may explain why sICAM-1:Fc administration resulted in reduced inflammatory foci when applied to NOD mice whereas CD40:Fc did not. Second, the level of CD40:Fc within the gland may not have been high enough to cause a significant effect. We found 10 ng/ml of CD40:Fc in the blood of treated mice, which is significantly higher than the levels in untreated mice and suggests higher levels locally in the SG. In transplantation rejection studies with blocking antibodies levels of 10–50 ug/ml were sufficient to prevent rejection, however it is unclear how this compares to our study in which CD40:Fc is continuously locally produced in the inflamed SG. Third, the CD40 inflammatory pathway in the SG may be redundant and blocking the interaction of CD40 with its ligand may be overtaken by other co-stimulatory pathways such as the CD80-CD28 interaction [31]. Combination therapies may therefore prove more successful. This has previously been shown in transplantation rejection experiments in which CD40 blockade was used in combination with CTLA4-IgG, which blocks CD80 and CD86. In this study, the combination of blocking both co-stimulatory pathways was 100 times more efficient than blockade of CD40 or CD80/86 alone [32]. This hypothesis is supported by the observation in our study that local CD40:Fc gene transfer did result in changes in the genes expression profile. Fourth, we cannot completely exclude the possibility that soluble CD40 may have led to stimulation of CD154 on cells expressing this ligand or that the human Ig1:Fc part of the construct bound and cross-linked Fc-receptors on the mouse (immune)cells, counteracting the effect of blockade of the cell-cell interaction between CD40 and CD154. Agonistic monoclonal antibodies to CD40 reduced collagen-induced arthritis, possibly through upregulation of IL-10 and to lesser extent IL-4 [33], indicating an anti-inflammatory role in addition to the pro-inflammatory role of CD40. In our study, although the immune response genes were mainly down-regulated, Phospholipase A2, group×(PLA2G10) was higher (4.3 fold) in CD40:Fc treated mice compared to controls. PLA2G10 upregulation was recently described in patients with SS [34]. Future studies using anergic monoclonal blocking antibodies could address whether this mechanism influences the effectiveness of treatment with CD40:Fc. Extra caution is necessary when considering the therapeutic use of sCD40:Fc. Therapy interfering with the CD40-CD154 with anti-CD154 antibodies has resulted in severe thromboembolic events [35]. This is possibly caused by ligation of CD154 on the platelet membrane [36]. The use of localized expression of CD40:Ig did not result in and thromboembolic events during this study suggesting this risk could be minimized.

In conclusion, despite the rather extensive literature supporting CD40 as a therapeutic target in autoimmune diseases, our study shows no clinical benefit of interfering with the CD40-CD154 interaction by expression of soluble CD40 fusion proteins at the levels of expression we were able to achieve in the SG in this animal model of SS. We observed changes in the gene profile after treatment, but this did not translate into reduced inflammation or improved SG function. This may suggest that the CD40-CD154 interaction alone is not as important in SS as previously suggested.

## Materials and Methods

### Vector Production

We used a plasmid for the murine CD40 coupled to the constant region of human immunoglobulin (IgG1:Fc), previously used for adenoviral vector gene transfer [10]. This gene was cloned into a recombinant adeno associated virus (AAV) plasmid containing a cytomegalovirus (CMV) promoter and the inverted terminal repeat (ITRs) sequences for AAV serotype 2 (AAV2). To generate recombinant AAV serotype 2 vectors (rAAV2), we used the adenoviral helper packaging plasmid pDG. Plates (15 cm) of ∼40% confluent 293 T cells were cotransfected with either pAAV-LacZ [37] or pAAV-CD40 according to standardized methods [38]. Clarified cell lysates were adjusted to a refractive index of 1.372 by addition of CsCl and centrifuged at 38,000 rpm for 65 hr at 20°C. Equilibrium density gradients were fractionated and fractions with a refractive index of 1.369–1.375 were collected. The titer of DNA physical particles in rAAV stocks was determined by quantitative (Q)-PCR and the vectors were stored at −80°C. The generated plasmid (pAAV2-CMV-mCD40-hFc) was transfected into HEK293 cells and secretion of the protein in the supernatant was measured by western blotting and an ELISA-kit for murine CD40 (R&D systems, Minneapolis, MN, USA). On the day of vector administration to NOD mice, the vector was dialyzed for 3 hr against saline.

### Animals, Vector Administration and Detection

Female NOD mice (Jackson Laboratory, Bar Harbor, ME, USA) were kept under specific pathogen-free conditions in the animal facilities of the National Institute of Dental and Craniofacial Research (NIDCR). The numbers of mice used in each experiment are indicated in the figure legends. Animal protocols were approved NIDCR Animal Care and Use Committee and the National Institutes of Health (NIH) Biosafety Committee. Vectors were delivered into the submandibular glands at 8, 10 and 16 weeks by retrograde instillation as previously described [30]. In short, female NOD mice were anesthetized with mild anesthesia (a combination of ketamine and xylazine) and 50 µl containing 1×10^11^ vector particles was administered to each submandibular gland by retrograde ductal instillation using a thin cannula (Intermedic PE10, Clay Adams, Parsippany, NJ, USA). To compare local versus distal treatment, one group of mice was injected in the right thigh muscle with 1×10^11^ vector particles at 10 weeks and was analyzed in a similar way as the locally treated mice. Mice were sacrificed at 16 or 20 weeks of age. At time of sacrifice, submandibular SGs were removed and cut in 4 equal parts. One part was homogenized and total genomic DNA was isolated using DNeasy blood & tissue kit (Qiagen, Venlo, the Netherlands). Vector was detected using Q-PCR and probes specific for promoter-gene sequences on an ABI StepOnePlus Real-Time PCR system (Applied Biosystems, Carlsbad, CA, USA). NOD mice develop diabetes independent of SS [39]. To treat hyperglycaemia and diabetes related dehydration a subcutaneous injection of long-acting Humalin N (1 U/mouse, every 24 hours; Eli Lilly, Indianapolis, IN, USA) was given to mice with blood glucose levels ≥250 mg/dL. In our facility, the incidence of diabetes is normally 50–70% by 20 weeks of age using this cut-off value.

### Histological Assessment and Immunohistochemistry

Two other parts of the SGs were cleaned of any adjacent (possibly lymphoid) tissue and cut into three cross-sectional parts. The first part was collected in formalin, embedded in paraffin and cut in 5 µm sections. Three sections (each 50 µm apart from the previous) were mounted on glass slides and stained with haematoxylin and eosin. The number of foci (where one focus is defined as an aggregate of ≥50 infiltrated cells per 4 mm^2^ [40] on each section) was counted by two different examiners who were blind to the assigned treatment group, and the mean focus score (FS) was determined. Sections were cut (7 µm) for staining with specific antibodies. The second SG part was emerged in Tissue-Tek optimal-cutting temperature (OCT) compound (Miles, Elkhart, IN, USA) and quickly frozen on dry ice. Sections (7 µm) were cut with a cryostat, mounted on glass slides and stored at −80°C until further use. For staining, sections were thawed and fixed in acetone for 10 minutes (for frozen sections) and deparaffinised and hydrated for paraffin embedded slides. Endogenous peroxidase was blocked, slides were washed in phosphate-buffered saline (PBS), and further blocked with 1% bovine serum albumin (BSA) and 10% normal goat serum (NGS; Dako, Glostrup, Denmark) in PBS overnight. The next day, slides were incubated with the following primary antibodies in 1% PBS and 5% serum of the host of the secondary antibody at room temperature for 1 hour: CD4, CD8, B220, CD19, CD11c, CD49b and CD68. Conditions used and information on the specific antibodies were previously published [2].

### Preparation of Salivary Gland Tissue Homogenates

The fourth SG part was snap-frozen on dry ice and stored at −80°C until further use. Prior to homogenisation, samples were thawed and kept on ice. Samples were crushed, placed in 2 mL tubes containing 1 mL HEPES lysis buffer (20 mM HEPES, 0.5 M NaCl, 0.25% Triton X-100 and 1 mM EDTA) and complete protease inhibitor (Roche, Mannheim, Germany), and lysed by shaking at 4°C overnight. The next day, samples were centrifuged at 1,500×g at 4°C for 10 minutes. Protein content of supernatants was measured using a bicinchoninic acid (BCA) protein detection kit (Pierce, Rockford, IL, USA) and stored at −80°C until further use.

### Saliva and Serum Collection

Saliva was collected 5 days before sacrifice. Saliva secretion was induced in anesthetized mice (see cannulation procedure) by subcutaneous (sc) injection of pilocarpine (0.5 mg/kg BW; Sigma-Aldrich, St. Louis, MO) and stimulated whole saliva was collected for 20 minutes (min) from the oral cavity by gravity with a hematocrit tube (Drummond Scientific Company, Broomall, PA, USA) placed into a preweighed 0.5 ml microcentrifuge tube. Saliva volume was determined by weight and expressed as µl/20 min*gram body weight. Serum was collected during sacrifice by heart puncture. Blood was left to clot on ice for 3 hours and centrifuged to collect serum.

### CD40:Fc Detection in Serum, Saliva, and Tissue Homogenates in ELISA

A commercially available mouse CD40 detection kit (Mouse CD40/TNFRSF5 DuoSet DY097, R&D systems) was used for detection in supernatant, serum, saliva and tissue homogenates. An adapted protocol was used for detection of the human Fc domain in the CD40 construct as follows. Plates were coated with the CD40 capture antibody and incubated with the samples as described in the protocol of the DY097 kit. After incubation with the samples, plates were washed three times with wash buffer and incubated with 100 µl of 1∶7500 anti human IgG-HRP (GTX 26759, Genetex, Irvine, CA, USA) for 1 hr. Plates were washed three times and incubated with substrate solution (R&D systems, Minneapolis, MN, USA) and stopped with 50 µl stop solution (R&D systems). The plates were read at 450 nm using a Spectramax M2 plate reader (Molecular Devices Corporation, Sunnyvale, CA, USA).

### CD40:Fc Detection in a Western Blot

Supernatant of CD40:Fc and mock transfected HEK293 cells was loaded (10×ug/lane) onto a Bis-Tris gel, after boiling for 5 minutes at a 1∶1 ratio with 2.5% beta mercapto/NU page LDS sample buffer (Invitrogen, Carlsbad, CA, USA), run and transferred to a nitrocellulose membrane. The blot was blocked with 5% milk powder in TBST buffer and incubated with biotin labeled anti-murine CD40 (R&D systems, MAB4401), followed by streptavidin-HRP (R&D systems) or anti human IgG-HRP (Genetex) and developed with ECL reagent (GE Heatlhcare, Buckinghamshire, UK). Anti-murine CD40 recognizes CD40 dimers well and monomers poorly (R&D product information).

### RNA Extraction, Amplification and Synthesis of Fluorescent cRNA

Total RNA was extracted from frozen SG samples using the RNeasy Mini Kit (Qiagen, Valencia, CA, USA) according to the manufacturer’s recommendations. The quality of RNA was measured with a 2100 Bioanalyzer (Agilent Technologies, Palo Alto, CA, USA), only samples with a 28S/18S ribosomal RNA ratio of 1.7 and RIN score >6.5 were used in the microarray study. The RNAs were amplified and labeled with a lower RNA input linear amplification kit (Agilent, Santa Clara, CA, USA). A total of 500 ng RNA was labeled with Cyanine 3-CTP according to the manufacturer’s instructions: Briefly, 500 ng of total RNA was first mixed with 2.0 µl of RNA spike (One-Color Spike, Agilent) previously diluted in a 1.5 ml tube including T7 primer and was the incubated at 65°C for 10 min. The reaction temperature was changed to 40°C after adding 8.5 µl of cDNA Master Mix reagents (Agilent) for 2 hours, and the samples were moved to a 65°C circulating water bath for an additional incubating for 15 min, and quench on ice for 5 min. Sixty µl of transcription Master Mix was added to the reaction including Cyanine 3-CTP (Agilent) for each sample for additional 5 hours at 40°C. The labeled and amplified cRNA was purified using an RNeasy Mini Kit (Qiagen). The cRNA was quantified by using a NanoDrop ND-1000 UV-VIS Spectrophotometer (version 3.2.1). Only cRNA with a total yield >1.65 µg/reaction and specific avidity >9.0 pmol Cy3 per µg cRNA were used in the following hybridization step.

### Microarray Hybridization and Data Extraction

Labeled cRNA was used to probe a 4×44 K microarrays (Agilent) containing 44 K mouse oligo probes. Microarrays were hybridized according to the manufacturer’s recommendations from One-Color Microarray-Based Gene Expression Analysis (Agilent). Briefly, each tube contained the following reaction reagents: 1.65 µg of new Cy3-labelled cRNA, 11 µl of 10x blocking agent, and 2.2 µl of 25x Fragment buffer. The mixture was incubated at 60°C for exactly 30 min and then 55 µl of 2×GE×Hybridization buffer was added to stop the fragmentation reaction. Following centrifugation 13.000 rpm for 1 min, 100 µl of the sample solution was loaded onto each array on the microarray slides that was then assembled in the hybridization chamber. The assembled slide chamber was then placed in a hybridization oven (Agilent) with rotating speed of 10 rpm at 65°C for 17 hours. After disassemble of the array hybridization chambers, were washed. Following the washing procedures, the slides were immediately scanned using a Microarray Scanner (Model: Agilent G2565AA System) to minimize the environmental oxidation and loss of signal intensities. The microarray data (tif images) file was extracted using the Agilent Feature Extraction (FE) (software version 9.5.1) program, for One-color gene expression, the default gene expression is specified in the FE grid template properties with selection of “GE1_QCM_Feb07” in this protocol. Only those chips that meet 9/12 of the report criteria were using in subsequent analysis.

### Pathway Analysis

Microarray analysis was performed using GeneSpring (GX 11) and included pre-processing raw-data, normalize data, QC samples/entities, t-test plus Benjamin correction, clustering, annotation and access biological context. Gene changes greater than 2 fold were used in the analysis. The comparisons of biological functional pathway or biomarkers were run by an IPA program (Ingenuity@ systems, Redwood city, CA, USA) or genego (metacore).

### Determination of Immunoglobulin Levels

IgG, IgA and IgM (Bethyl Laboratories, Montgomery, TX, USA) were measured by commercially available ELISA kits according to the manufacturer’s protocol. Values were corrected for total protein content in the SG protein homogenates.

### Statistical Analysis

Differences between experimental groups were assessed using the non-parametric Wilcoxon’s rank-sum test or parametric Student’s t-test depending on data distribution. All analyses were performed with GraphPad Prism statistical software (GraphPad Software Inc. version 5.01, La Jolla, CA, USA). A P value ≤0.05 was considered to be statistically significant.
